# Use, Validity and Reliability of Inertial Movement Units in Volleyball: Systematic Review of the Scientific Literature

**DOI:** 10.3390/s23083960

**Published:** 2023-04-13

**Authors:** Diego Hernán Villarejo-García, Adrián Moreno-Villanueva, Alejandro Soler-López, Pedro Reche-Soto, José Pino-Ortega

**Affiliations:** 1 Faculty of Sports Sciences, University of Murcia, 30100 Murcia, Spain; dvillarejo@um.es (D.H.V.-G.); pedrojose.reche@um.es (P.R.-S.); 2 Faculty of Health Sciences, Isabel I University, 09003 Burgos, Spain; adrian.moreno@ui1.es; 3 BIOVETMED & SPORTSCI Research Group, Department of Physical Activity and Sport, Faculty of Sport Sciences, University of Murcia, 30100 Murcia, Spain

**Keywords:** inertial measurement units, jump height, sports technology, micro-electro-mechanical systems, sensors, performance

## Abstract

The use of inertial devices in sport has become increasingly common. The aim of this study was to examine the validity and reliability of multiple devices for measuring jump height in volleyball. The search was carried out in four databases (PubMed, Scopus, Web of Sciences and SPORTDiscus) using keywords and Boolean operators. Twenty-one studies were selected that met the established selection criteria. The studies focused on determining the validity and reliability of IMUs (52.38%), on controlling and quantifying external load (28.57%) and on describing differences between playing positions (19.05%). Indoor volleyball was the modality in which IMUs have been used the most. The most evaluated population was elite, adult and senior athletes. The IMUs were used both in training and in competition, evaluating mainly the amount of jump, the height of the jumps and some biomechanical aspects. Criteria and good validity values for jump counting are established. The reliability of the devices and the evidence is contradictory. IMUs are devices used in volleyball to count and measure vertical displacements and/or compare these measurements with the playing position, training or to determine the external load of the athletes. It has good validity measures, although inter-measurement reliability needs to be improved. Further studies are suggested to position IMUs as measuring instruments to analyze jumping and sport performance of players and teams.

## 1. Introduction

The use of inertial motion units (IMU) has enabled sport scientists, coaches and athletes to obtain physiological, kinematic and spatial positioning data [1,2]. These data provide locomotor variables (e.g., distance, number of sprints, player load) [3], movement variables (e.g., velocity, acceleration) [4] and sport-specific patterns (e.g., player skills) [5]. These variables have been used to improve physical performance [6], monitor technical and tactical performance [7] and improve the injury prevention and recovery processes [8]. The main advantages of this type of technology may lie in its application in the real-life sport context [9], ease of use [10], large volume of stored information [11], real-time monitoring [12] and large data diversity [13]. In team sports, the data collected by these devices have generally focused on describing efforts to which athletes are subjected in competition and training [14]. To detail these efforts, the description of movement patterns [15], specific movements [16] or load indices extracted from the movements performed [17] have been used. The most analyzed variables have been heart rate [18], distances covered [19], speed of movements [20] and relative intensities [21]. In indoor team sports, the use of these variables has been mainly to describe training load [22].

In volleyball, the training and competition load alternates low and high intensity actions [23]. Of these actions, jumping is the most frequent high-intensity effort [24]. Therefore, training in this sport should have, as one of its objectives, the development of jumping as a specific capacity [25]. Thus, the assessment of jumping can provide significant information on the sporting and clinical needs of athletes [26]. Field and laboratory tests have been used for the assessment of jumps [27]. The use of technology has further refined jump analysis, primarily using video analysis methodologies [28] and biomechanical analysis [29]. These jump analysis methodologies allow for an accurate determination of height, velocity, force exerted and even joint angle parameters. For these variables, few systems report data in real time and from several athletes simultaneously.

With the development of IMUs, it has been possible to use this technology to measure the height and frequency of jumps [30]. In a systematic review by Clemente [30], the validity and reliability of IMUs to measure jumping in controlled and laboratory situations was tested. However, this review did not analyze the application of IMUs as a function of sport, and concluded that of the sixteen devices that submitted reliability and validity tests, only seven showed acceptable results. The analysis of validity and reliability in ecological settings is essential, as one of the main uses of IMUs is in sporting contexts. This type of research is important to determine how accurately and precisely aspects of sport performance can be quantified using IMUs and to what extent their use is justified. Therefore, although previous research studies have assessed the validity and reliability of IMUs for measuring jumping, these investigations were usually conducted in controlled laboratory situations and did not specify the type of sport. Although information on the use of IMUs in volleyball is limited, their usefulness is evident. Therefore, the aim of this study was to examine the validity and reliability of multiple devices for measuring jump height in volleyball, determining the degree of validity and reliability of these devices commonly used by coaches. The specific objectives of this research were: (a) to systematically identify scientific publications that have used IMUs as assessment devices in volleyball and (b) to analyze the use, validity and reliability of IMUs in this sport.

## 2. Method

This manuscript is a systematic review [31] on peer-reviewed, scientific papers related to the use, validity and reliability of IMUs used to assess performance in volleyball. The Web of Science (Web of Science Core Collection, MEDLINE, Current Contents Connect, Derwent Innovations Index, KCI-Korean Journal Database, Russian Science Citation Index and Scielo Citation Index), PubMed, SPORTDiscus and Scopus electronic databases were searched on 30 December 2022 using the keywords “volleyball” and “accelerometry” or “accelerometer” or “accelerometer” or “gyroscope” or “inertial” or “sensor” or “wearable” or “measurement unit” or “wearable system” or “device” or “IMU” or “MEMS” or “microelectromechanical” and “jump” or “activity profiles” or “specific movements”. The bibliographic reference lists of the included studies were also reviewed to identify studies likely to be included in the analysis that had not appeared through our search strategy. This review process of the reference lists was also performed with the article extracted from external sources. Any disagreements were resolved by consensus between two investigators (A.M.V and A.S.L.) and arbitration by a third investigator (J.P.O.).

**Figure 1:**
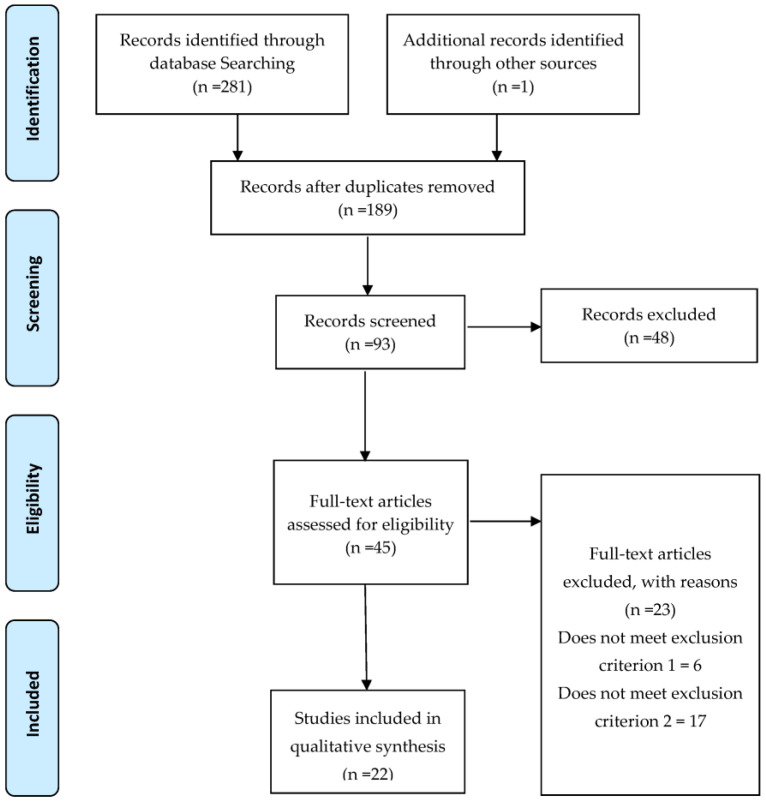
PRISMA flow diagram.

One investigator (A.S.L.) was in charge of conducting the electronic searches, identifying relevant studies and extracting the data in a standardized and non-pooled manner. A systematic review was performed according to PRISMA (preferred reporting items for systematic reviews and meta-analyzes) guidelines [32] and guidelines for conducting systematic reviews in sport science [33] (Figure 1).

In the present review, the inclusion criteria for these articles were: (1) the sample includes only volleyball athletes of any level, age and gender; (2) IMUs used for data collection; and (3) original articles only from the field of sport sciences. All included studies were deemed to have had appropriate ethical approval by a competent review committee. Studies were excluded if: (a) the sample involved other athletes in addition to volleyball players; (b) they used other types of devices than IMUs; and (c) the type of document was a review, letter to editors, trial registration, proposal for protocols, editorial, book chapter and conference abstract, or any document not related to the field of sport sciences.

### 2.1. Data Extraction and Analyzed Variables

The Cochrane Consumers and Communication Review Group’s data extraction protocol [34] was used to group four characteristics of the studies: (a) methodological characteristics, (b) substantive characteristics, (c) validity characteristics, and (d) reliability characteristics.

Results describing “methodological” characteristics detailed: modality, design, subjects, level, gender and age, commercial IMUs’ name, technical characteristics, context of the studies and variables analyzed. The “substantive” characteristics detailed: quality, objectives, results and applications. “Validity” characteristics described: context, criterion instrument, variables identified, validated instrument, statistical analysis and value. “Reliability” characteristics detailed: criterion instrument, validated instrument, logarithm, measured variables, statistical analysis and value. First, one researcher (A.S.L.) extracted the data from the included studies and a second researcher (A.M.V.) then checked the extracted data. Disagreements were resolved by consensus.

### 2.2. Quality of the Studies

Two authors (A.M.V. and A.S.L.) were in charge of analyzing the risk of reporting bias of the selected studies, using an adapted version of the STROBE evaluation criteria, as other studies such as the one by O’Reilly et al. [35]. Following this evaluation methodology, each article was evaluated using 10 specific items (exposed at the bottom of Table 1). In the event of any disagreement in the evaluation of any study and/or item, it was discussed and resolved by consensus between the two previously cited authors. The study rating was interpreted qualitatively following O’Reilly et al. [35]: from 0 to 7 points, the study was considered of low quality, while, if the study was rated from 8 to 10 points, the article was considered to be of high quality.

The methodological risk of bias was assessed using the methodological index for nonrandomized studies (MINORS) by two authors (A.M.V. and A.S.L.) [55]. MINORS comprises twelve items, four of which are only applicable to comparative studies. Each item is scored 0 when the criterion is not reported in the article, 1 if it is reported but not sufficiently met, or 2 when it is adequately met. Higher scores indicate a good methodological quality of the article and a low risk of bias. Therefore, the highest possible score is 16 for non-comparative studies and 24 for comparative studies. MINORS has provided acceptable inter- and intra-rater reliability, internal consistency, content validity, and discriminant validity [55,56].

## 3. Results

### 3.1. Identification and Selection of Studies

The process of search, identification and selection of studies is illustrated in Figure 1.

### 3.2. Methodological Quality

The overall reporting risk of bias of the cross-sectional studies can be found in Table 1.

The results of the methodological risk of bias of the articles included in this review can be found in Table 2.

Table 3 shows the methodological characteristics of the studies. Of the 22 studies found, 19 studies (90.47%) correspond to the indoor modality and only 3 (9.53%) to the beach modality. The number of subjects who participated in the studies and who used IMUs in competition, training or evaluation sessions ranged from 5 subjects [44] to 115 subjects [36].

Regarding the level of the participants, the most characteristic sample was of elite (90.47%) and local level (9.37%) teams. The age of the participants was between 16.1 and 27.6 years and 57.17% of the studies assessed men, 23.77% worked with women and 19.06% assessed both sexes.

Six devices were identified (Vert, Catapult, Sunto, Shimmmer, Blast and Zephyr BioHarness). The most widely used was the Vert device (57.14%). This may be because the Vert device is designed and marketed specifically for volleyball [10], whereas the other devices can be adapted to any sport. In terms of sensor placement, the results show that 80.95% (n = 17) were placed in the central area of the athlete’s body, although with differences, as they were placed in the iliac crest [10,24,36,37,38,45,47,49,51], sternum [24,40], lumbopelvic [41,48], thoracic vertebrae and scapula [42,43,52,54]. Only 9.52% (n = 2) used the lower extremities, in metatarsals and tibia.

Regarding the objectives of the studies, 52.38% focused on determining the validity and reliability of the IMUs. A total of 28.57% focused on controlling and quantifying the external load and 19.05% on describing differences between playing positions. The studies that aimed to compare the jumps recorded by the IMUs with variables such as internal load [45,54], playing position [24,25,44], training [44] and/or match play [25] are presented in Table 4.

In this review we found seven studies [10,24,39,41,47,50,51] that used a criterion of concurrent validity of measures. To do so, they compared the data collected by the device with data collected visually. This visual inspection was performed by one [39,41,47,50] or two expert judges [10,24,51] and all these studies were conducted retrospectively.

Visualization was performed in ecological training and match-context [10,24,39,47,51] training [10,39], structured practices [41,47,50] and only one laboratory study [50]. Some studies determined the reliability of IMUs for differentiating jumps from other types of displacements [10,24,39,41,47].

Table 5 shows the results of the validity characteristics of the studies.

We found 10 studies [10,24,36,37,39,46,47,48,50,51] that examined the reliability of the measures.

As for the commercial device that underwent the most validity testing, the results of this review indicate that it was the Vert device [10,24,36,37,47,51]. Overall, this device was found to be reliable for measuring jump height. When this device was compared to a video camera motion analysis system [10], it showed good to excellent correlations (0.879–0.998).

Table 6 shows the results of the reliability characteristics of the studies.

## 4. Discussion

The objectives of this study were: (a) to systematically identify the scientific publications that have used IMUs as assessment devices in volleyball and (b) to analyze the use, validity and reliability of IMUs in this sport. 

### 4.1. Use of IMUs

Methodological Characteristics

Regarding the use of IMUs, a predominance in indoor environments compared to outdoor scenarios stands out. These results follow the same trend as other research conducted in volleyball that focused on analyzing other variables such as injuries [57] and training methods [58]. This may be because beach volleyball is a novel modality compared to indoor volleyball, and its body of knowledge does not reach similar volumes. Associated with this, a greater popularity of indoor volleyball and thus a greater number of participants may justify more interest and evidence in this modality. The use of IMUs, however, may be equally beneficial in both modalities. In turn, the number and characteristics of the participants in which IMUs were used are quite heterogeneous. In terms of age, few studies evaluated juveniles. Additionally, no studies were found in children. The use of IMUs in youngsters and children could be very useful, as it would provide information on the characteristics of competition in formative stages, which would help to adapt training methods. Gender differences can be explained by the bias that exists in science [59]. The use of IMUs would serve women and men equally well, and a gender comparison of the data could help to understand similarities and differences in performance and physical demands.

Regarding the level of the teams evaluated, the use in elite teams stands out. For most of the studies, data acquisition has been performed both in training and in competitions [24,25,38,39,42,43,44,45,49,53,54], laboratory [36,37,46] and structured practices [50]. Some authors have combined these contexts depending on the aims of the studies [10,41,47,51]. In controlled laboratory conditions, it is easier to standardize protocols and performances; however, in sport settings, real data are obtained from the demands of competition.

On the technical aspects of IMUs, the results show that the most commonly used type of inertial sensor was 3D accelerometers. Regarding the variety of devices available on the market, four were identified (Vert, Catapult, Sunto, Shimmmer, Blast and Zephyr BioHarness). The most widely used (Vert) is a device specifically designed and marketed for volleyball [10], which justifies its recurrence among the studies. The other devices used can be adapted to any sport and provide data on mechanical and functional capacities, as well as external loading.

Regarding the placement of the sensors, the central area of the body is the most used, specifically the iliac crest, the sternum, the lumbopelvic area, thoracic vertebrae and scapula, and a few in the ankles and tibia. The iliac crest is a body area where the use of these devices has been most validated for volleyball [10,25,36,37,38,44,45,47,49,51,53]. One possible reason is that since the devices are designed for jumping quantification, the iliac crest represents a central body area of the body, and therefore concentrates much of the athlete’s mass. Previous studies have validated devices placed on the iliac crest by comparing them with values obtained in CMJ tests [24]. The placement of IMUs should not be a limitation of movement or discomfort for athletes. In fact, the use of the device on the back, near the scapulae, provides security as it prevents the device from detachment and even minimizes the risk of injury to the athlete [54].

Finally, the variables collected in the majority of studies were jump count (77%, n = 17) and height (63%; n = 14). Thirty-two percent (n = 7) of the studies combined variables derived from count, height and time. Two studies used algorithms to express external load indices [24,54]. In this sense, the monitoring of jumps in volleyball seems to be an indicator of the greatest interest for coaches. The monitoring of jumps provides relevant data for coaches to control the training load and the athlete’s performance [60,61]. However, it is debatable whether jump count and height are sufficient estimators to understand the training load. In this regard, algorithms have been proposed that combine count, height, travel speed and athlete mass [24]. In some devices, these load indices have acceptable validity and reliability as a measure of load [62]. However, due to the specific characteristics of volleyball and each playing position, they must be specifically validated. Additionally, and due to the individual characteristics of each athlete, they should be combined with internal load measures [45,54] to have a more accurate value of the load to which the athlete is subjected.

### 4.2. Substantive Characteristics

The aims of the studies focused on three aspects: determining the validity and reliability of IMUs (52.38%), monitoring and quantifying external load (28.57%) and describing differences between playing positions (19.05%). In other field sports [30,63,64], the use of IMUs has principally focused on monitoring training load, detecting risks of overtraining and assessing sport performance. For example, the use of devices to monitor external load has shown a positive relationship with internal load in training and competition [42,43]. Thus, Lima et al. [45] have found high relationships between number of jumps and RPE. It has also been useful to monitor performance during matches by controlling the number of jumps between sets [25,44]. The use of IMUs has allowed the identification of differences between playing positions in terms of the number of jumps and the height reached in the jumps [44,49]. The middle blocker recorded the highest number of jumps, while the setter recorded the lowest number [49]. Furthermore, as observed in the study by Bahr et al. [65], there are also sex differences in the total number of jumps recorded during training and matches in young elite volleyball players. All of the above shows possible practical applications to determine and individualize the training load and to use this information to improve performance and control the risk of injury.

### 4.3. Validity of IMUs in Volleyball

Studies that examine the validity of IMUs are important as they reflect the degree to which an instrument is representative of the variable it is intended to measure. The most commonly used criterion of validity was concurrent [10,24,39,41,47,50,51]. The comparison of data obtained by technological devices and visualization data is a widely used technique for device validation. In this sense, studies by McDonald et al. [47], Gageler et al. [39] and Charlton et al. [10] correctly identified 97–99% of volleyball-specific jumps in comparison to other movements (e.g., displacements, hits, serves, etc.). This method compares the frequency of jumps detected by visual inspection and IMU (true positives), records detected by visual inspection but not by IMU (true negative) and records not detected by visual inspection but detected by IMU (false positive). In all studies, the percentage of true positives was above 95% [10,39].

Only the study by Jarning et al. [41] did not differentiate jumping in the serve and smash from other movements. This may be because only acceleration data were used and the algorithm used did not allow differentiation. Regarding the types of jumps observed by visual inspection and counted by IMUs, a comparison between studies is difficult as the definitions of jumps are different (e.g., Charlton et al. [10] and McDonald et al. [47]) and in the studies where specific jumps were observed (e.g., spike, block, serve, etc.), the definitions were not found. The absence or differences in the operational definitions of the actions that are observed and quantified is one of the main problems to be solved in future research with the aim of providing greater logical and content validity [66], as well as precisely defining the variables collected, describing the reliability of the observations in the visual inspection and explaining the data control process [67].

### 4.4. Reliability of IMUs in Volleyball

As in the validity studies, the criterion for establishing the reliability of the IMUs and determining accuracy was to compare them with data obtained with a gold-standard instrument and to analyze the agreement between them. The results of this systematic review indicate that the characteristics of these instruments used as criteria for measurement comparison ranged from mechanical use, such as the Vertec [24,36,48] force platforms [24,39,50], video camera analysis systems [10,47], or other IMUs [10,24,37]. In this sense, the criterion instrument should present evidence of proven reliability, which makes the use of Vertec and other IMUs as “gold standard” criterion instruments cautious [68,69].

In terms of the statistical techniques used to establish the agreement of the measurements, the Pearson’s correlation coefficient (r) stands out. In the study by MacDonald et al. [47], strong correlations were also observed between Vert and a 3D-motion analysis video system (r: 0.88–0.89) and narrower limits of agreement (−6.1 to 9.8 cm). This may be because this work used a laboratory jumping protocol (CMJ) and elite athletes. However, this work underestimated the maximum jump height by 2.5 cm compared to the reference method. The authors of this study (MacDonald et al. [47]) stated that Vert did not find small changes in performance given the standardized standard errors. In this sense, the susceptibility of the devices should be able to identify small changes in jump height. In a more recent study [51], which compared the results of the Vert device with data obtained on a force platform, similarities were found to that which was reported by Charlton et al. [10] and Mc Donald et al. [47], whereby a mean error of 3.02–3.13 cm and limits of agreement of 7.65–6.60 were found. Vert has utility for quantifying jumping load during training and competition in volleyball, but further studies are needed to make generalizations regarding the use of Vert to assess changes in jumping performance [30].

However, regarding the use of Pearson’s correlation coefficient used in some studies [10,24,36,39,47], this statistical analysis is not the most appropriate for determining agreement between devices. In fact, the intra-class correlation coefficient presents characteristics that make them a better estimator (e.g., Schmidt et al. [51]; Damji et al. [37]; Markovic et al. [46]; Schleitzer et al. [50]; Montoye et al. [48]). Additionally, Bland–Altman statistics are highlighted as a means to analyze the limits of agreement between devices (e.g., Schmidt et al. [51]; Damji et al. [37]; Markovic et al. [46]; Schleitzer et al. [50]; Montoye et al. [48]). It is important to include in the statistical analysis the calculation of the minimum detectable change (e.g., Skazalski et al. [24]) as an estimator of the minimum degree of difference to determine whether there are differences between the two measuring instruments [70]. It would therefore be desirable for a reliability analysis to include the calculation of a set of statistics intended to provide information on the level of agreement and the magnitude of errors.

In general, the results of studies which analyze the reliability of measurements show that devices have a measurement error in quantifying jump height, in some cases overestimating [24], and in others underestimating [10,24,47]. These differences may be due to the methodology and instruments used to measure jump height. Therefore, it is difficult to make comparisons between studies, as many of them do not explain the method to establish vertical displacement, and thus detect possible systematic measurement errors. Of all the studies found, only three [39,46,50] detail the logarithm used to calculate the jump distance. It is understandable that commercial brands do not disclose the mathematical calculations for estimating this height. However, knowledge of these would help to understand one of the possible causes of technological error. Specifically, it would help to know the systematic error of the measurement and its possible solutions.

However, studies suggest that the devices have high sensitivity for detecting jumps, albeit with significant errors. These errors can be significant when the aim is to detect changes in athletes’ performance. However, if the measurement error is known, the use of devices provides benefits in real environments [71] without losing utility.

## 5. Conclusions

In general, it can be concluded that the studies conducted in volleyball using IMU devices have aimed to validate and measure the reliability of these devices for counting and measuring vertical displacements and/or comparing these measures with the playing position, training or determining the external load of the athletes. Validity measures for jump counting have been shown to be good to excellent, while reliability measures for height estimation have shown conflicting data. When the devices are used in real-world settings, they have proven to be reliable tools for quantifying and individualizing training load. 

## 6. Limitations of the Paper and Future Approaches

In addition to this research, knowledge with regard to the magnitude and direction of the applied force is also important in volleyball. Usually, a combination of multiple (two or three) uniaxial accelerometers with IMU is used to detect these variables, but their high cost, size and total system cost of complexity increase their difficulty to be used routinely [72,73]. Future research could focus on developing the reliability and validity of these variables.

The study’s findings highlight the relevance of considering the recording system to analyze the kinematic data in volleyball, especially among senior players. The use of IMUs in the youth and children could be very useful, as it would provide information on the characteristics of competition in formative stages, which would help to adapt training methods.

## Figures and Tables

**Table 1:** Reporting risk of bias assessment of the included studies.

Study	1	2	3	4	5	6	7	8	9	10	Quality
Borges, 2017 [36]	1	0	0	1	1	1	1	0	0	0	Low
Charlton et al., 2017 [10]	1	0	1	1	1	1	1	0	0	0	Low
Damji, 2021 [37]	1	0	1	1	1	1	1	1	1	0	High
de Leeuw, 2022 [38]	1	1	1	1	1	1	1	0	0	1	High
Gageler, 2015 [39]	1	0	1	1	1	1	1	1	1	0	High
Gielen, 2022 [40]	1	0	1	1	1	1	1	0	0	0	Low
Jarning, 2015 [41]	1	0	1	1	1	1	1	0	0	1	Low
Joao, 2021 [42]	1	0	1	1	1	1	1	1	1	0	High
Kupperman, 2021 [43]	1	0	1	1	1	1	1	1	0	1	High
Lima, 2019a [25]	1	1	0	1	1	1	1	0	1	1	High
Lima, 2019b [44]	1	0	1	1	1	1	1	0	1	1	High
Lima, 2020 [45]	1	1	1	1	1	1	1	1	0	1	High
Markovic, 2021 [46]	1	0	0	1	1	1	1	0	0	1	Low
McDonald, 2017 [47]	1	1	1	1	1	1	1	1	1	0	High
Montoye, 2018 [48]	1	1	1	1	1	1	1	1	1	0	High
Piatti et al., 2022 [49]	1	1	1	1	1	1	1	1	0	0	High
Schleitzer, 2022 [50]	1	0	1	1	1	1	1	1	1	1	High
Schmidt, 2021 [51]	1	0	1	1	1	1	1	0	0	1	Low
Setuain, 2021 [52]	1	0	1	1	1	1	1	1	1	0	High
Skazalski, 2018 [24]	1	0	1	1	1	1	1	1	1	0	High
Skazalski, 2018b [53]	1	1	0	1	1	1	1	1	1	0	Low
Vlantes, 2017 [54]	1	1	1	1	1	1	1	1	1	0	High

Note: (item 1): provide an informative and balanced summary of methods conducted and the main findings (item 1); establish specific objectives and hypotheses (item 2); indicate the inclusion/exclusion criteria, as well as the sources and methods of selection of the participants (item 3); provide data sources and details of the evaluation methods for each variable of interest; describe the comparability of the evaluation methods (item 4); explain how quantitative variables were used; describe and justify which groups were chosen (item 5); expose the characteristics of the study participants (item 6); summarize the key results in a manner consistent with the objectives of the study (item 7); analyze and exposes the limitations of the study; discuss both the direction and the magnitude of any potential bias (item 8); offer a cautious interpretation of the results (item 9); and indicate the source of funding and the role of the funders of this study and (item 10).

**Table 2:** Methodological risk of bias assessment using MINORS checklist.

Study	1	2	3	4	5	6	7	8	9	10	11	12	Score
Borges, 2017 [36]	2	2	1	2	2	2	2	-	-	-	-	1	14/16
Charlton et al., 2017 [10]	2	2	1	2	2	2	2	-	-	-	-	1	14/16
Damji, 2021 [37]	2	2	2	2	1	2	2	-	-	-	-	2	15/16
de Leeuw, 2022 [38]	2	2	2	2	1	2	2	-	-	-	-	2	15/16
Gageler, 2015 [39]	2	2	2	2	1	1	2	0	2	2	1	1	18/24
Gielen, 2022 [40]	2	2	2	2	1	2	2	0	2	1	2	2	20/24
Jarning, 2015 [41]	2	2	1	1	0	2	2	0	2	2	1	2	17/24
Joao, 2021 [42]	1	2	2	1	2	1	2	-	-	-	-	1	12/16
Kupperman, 2021 [43]	2	2	2	2	0	2	2	0	2	0	1	1	16/24
Lima, 2019a [25]	1	2	1	1	2	1	2	0	2	1	0	1	14/24
Lima, 2019b [44]	2	2	2	2	1	0	2	-	-	-	-	1	12/16
Lima, 2020 [45]	2	2	2	2	1	2	2	-	-	-	-	2	15/16
Markovic, 2021 [46]	2	2	2	2	1	2	2	-	-	-	-	2	15/16
McDonald, 2017 [47]	2	2	2	2	1	2	2	-	-	-	-	1	14/16
Montoye, 2018 [48]	2	2	2	2	1	1	2	-	-	-	-	1	13/16
Piatti et al., 2022 [49]	2	2	2	2	1	1	2	0	2	2	2	2	20/24
Schleitzer, 2022 [50]	2	2	2	2	0	1	2	-	-	-	-	2	13/16
Schmidt, 2021 [51]	2	2	2	2	1	1	2	0	2	2	1	1	18/24
Setuain, 2021 [52]	2	2	2	2	1	2	2	-	-	-	-	1	14/16
Skazalski, 2018 [24]	2	2	2	1	0	2	2	-	-	-	-	2	13/16
Skazalski, 2018b [53]	1	2	2	2	0	1	2	-	-	-	-	1	11/16
Vlantes, 2017 [54]	2	2	2	2	1	2	2	-	-	-	-	2	15/16

Note: The MINORS checklist asks the following information (2 = High quality; 1 = Medium quality; 0 = Low quality): clearly defined objective (item 1); inclusion of consecutive patients (item 2); information collected retrospectively (item 3); assessments adjusted to objective (item 4); evaluations carried out in a neutral way (item 5); follow-up phase consistent with the objective (item 6); dropout rate during follow-up less than 5% (item 7); a control group having the gold standard intervention (item 8); contemporary groups (item 9); baseline equivalence of groups (item 10); prospective calculation of the sample size (item 11); and appropriate statistical analysis (item 12).

**Table 3:** Methodological characteristics of the studies found.

Study	Modality	Subjects	Level	Sex/Age	IMU	Placement	Context	Variables
Borges, 2017 [36]	Indoor	112	Brazilian National team	M/17.8	Vert Classic	Iliac crest	Laboratory	Height
Charlton et al., 2017 [10]	Indoor	18	Elite junior	M/16.94	Vert Classic	Iliac crest	Training Laboratory Competitions	Frequency Height Derivatives
Damji, 2021 [37]	Indoor	14	University of Canada	M-F/20.9	G-Vert Shimmer3	Iliac crest	Laboratory	Landing impacts
de Leeuw, 2022 [38]	Indoor	14	International level	M/27	G-Vert	Iliac crest	Competition Training	FrequencyHeight
Gageler, 2015 [39]	Indoor	12 7	National level	M-F/16.20	GPSports Systems	T10 vertebra	Competition Training Laboratory	Frequency Flight times
Gielen, 2022 [40]	Indoor	8	Belgian first and second division	M/19.75	Zephyr BioHarness 3.0	Sternum	Competition	Jumps, accelerations and FC
Jarning, 2015 [41]	Indoor	12	Norwegian National team	M/22.5	ActiGraph GT3X+	Lumbosacral vertebra	Structured practice	Acceleration Frequency Displacements
Joao, 2021 [42]	Beach	12	Professionals Portugal	F/27.6	Minimax S4, Catapult	C7 and T2 vertebrae	Competition	Distances, meters, speed, acceleration /desac, jumps, Derivatives
Kupperman, 2021 [43]	Indoor	11	División I NCAA, USA	F/19.36	Clearsky T6; Catapult	Between scapulae	Competition Training	Distances, meters, speed, accel /desac, jumps, COD, Derivatives
Lima, 2019a [25]	Indoor	7	Portuguese First Division Professionals	M/26.7	Vert Classic	Iliac crest	Competition	Frequency Height Derivatives
Lima, 2019b [44]	Indoor	5	Portuguese First Division Professionals	M/26.7	Vert Classic	Iliac crest	Training	Frequency Height Derivatives
Lima, 2020 [45]	Indoor	8	Portuguese First Division Professionals	M/23.0	Vert Classic	Iliac crest	Training	Frequency Derivatives
Markovic, 2021 [46]	Indoor	13	Serbia National Team	F/24.6	LSM6DS33	Metatarsus	Laboratory	Height
McDonald, 2017 [47]	Indoor	13	Elite Calgary, Canada	M/16.1	Vert Classic 2.0	Iliac crest	Laboratory Structured practice Competition	Frequency Height Displacements
Montoye, 2018 [48]	Indoor	20	NCAA Division III Varsity University	F/18.9	Blast Athletic Performance Modelo B0113	Lumbosacral vertebra	Structured practice	Height
Piatti et al., 2022 [49]	Indoor	12	Elite	M/25.8	Vert	Iliac crest	Competition Training	Frequency Height
Schleitzer, 2022 [50]	Beach	20 5	Students Regional level	M-F/-	Suunto movesense original	Sternum Malleolus	Laboratory Structured practice	Frequency Height
Schmidt, 2021 [51]	Beach	8 11	German National Team different levels	F/18.4 M/24.3	Vert TM Classic	Iliac crest	Competition Laboratory	Frequency Height
Setuain, 2021 [52]	Indoor	12	Brazilian first division	M/23.7	Vert Classic	TThird lumbar vertebra	Training	Jump Biomechanics Strength
Skazalski, 2018 [24]	Indoor	13 8 22	Qatar First Division Recreational	M/adults	Vert Classic	Iliac crest Sternum Tibia	Competition Structured practice	Frequency Height
Skazalski, 2018b [53]	Indoor	14	Qatar First Division	M/adults	Vert Classic	Iliac crest	Competition Training	Frequency Height
Vlantes, 2017 [54]	Indoor	11	NCAA Division I	F/19.99	Catapult Optimeye S5	Between scapulae	Competition	Frequency Height Derivatives

**Table 4:** Substantive characteristics of the studies found.

Study	Aim of the Study	Relevant Results	Applications of IMUs
Borges, 2017 [36]	Determine IMU reliability	Differences in attacking jumps 70.9 ± 8.2 and 76.3 ± 7.5 cm (r = 0.75); Differences in blocking jumps 53.7 ± 6.1 and 58.5 ± 5.7 cm (r = 0.75); IMU overestimation of the attack (7.1%) and blocking (8.2%) jumps.	Caution in assessing specific jumps.
Charlton et al., 2017 [10]	Determine validity and reliability of IMU	High correlation between devices IMUs (r = 0.83–0.97); Differences between devices and motion analysis (3.57 and 4.28 cm); Lack of accuracy for height measurement Accuracy for counting 0.998 (0.995–1.000%).	Usefulness for external training load control; caution for evaluating jumps; algorithm proposal to quantify external training load.
Damji, 2021 [37]	Determine reliability for measuring landing impacts between IMUs	Low concordance values (−84.13% and 52.37%) and high bias between IMUs (average bias of −15.88%).	Caution to control external training load, taking into account landings.
de Leeuw, 2022 [38]	Identify and correlate injury risks through external load and wellbeing indicators in a season	70% of players indicating “difficulty in training” were related to jumping loads; high differences between players.	Caution to use jumping frequency as a predictor of injury if thresholds are not individualized.
Gageler, 2015 [39]	Determine validity and reliability of IMU for counting jumps	99% of jumps were identified;Underestimated flight times (0.015 s ± 0.058 s).	Useful for control and individualization of external load; caution in assessing heights.
Gielen, 2022 [40]	Determine the relationship between internal and external load over the course of a season	Significant correlations between maximum accelerations and maximum HR in the warm-up jumps (*p* = 0.62/0.49) not significant in the game; high correlation between activity and average HR in matches (*p* = 0.67).	Usefulness for external load control; caution with the relationship between external and internal load.
Jarning, 2015 [41]	Determine whether acceleration measured with accelerometer identifies jumps	The service serve and the smash could not be distinguished as movements without jumping (*p* = 0.422 and 0.999).	The methodology used is not useful for skip counting.
Joao, 2021 [42]	Quantifying the external load of players	Difference between playing positions in external load parameters (*p* = 0.000) and in jump height between sets (*p* = 0.004).	Usefulness for external load and fatigue monitoring in competition.
Kupperman, 2021 [43]	Quantify external and internal load in a season and describe differences between playing positions	High correlation between RPE and IMU data (*p* ≤ 0.001); Significant differences in IMU data between playing position (*p* ≤ 0.001/>0.004).	Usefulness for monitoring and individualization of training load and fatigue.
Lima, 2019a [25]	Describe jumps in playing positions and sets	Difference between positions and types and intensities of jumping; No differences in heights between sets.	Usefulness to control and individualize the external training load.
Lima, 2019b [44]	Describe load, playing positions and microcycle	Setter jumps more than middle blockers and outside hitters; Differences within the microcycle.	Usefulness to control and individualize the external training load.
Lima, 2020 [45]	Comparing internal and external load	Positive relationship between RPE and number of jumps (r = 0.17).	Usefulness for external training load and fatigue monitoring.
Markovic, 2021 [46]	Determine validity and reliability of IMU	High levels of validity for estimating jump height (CMJ t = 0.897, *p* = 379; ICC = 0.975; SQJ t = 0.564, *p* = 0.578; ICC = 0.921) and reliability (ICC > 0.872).	Usefulness for assessing jump heights.
McDonald, 2017 [47]	Determine validity and reliability of IMU	Overestimation of count in competition; High sensitivity in practice (96.8%); Underestimated height (2.5 to 4.1 cm).	Usefulness for external training load control; caution for measuring jumps and counting jumps in training and competition by the minimum threshold of 15 cm.
Montoye, 2018 [48]	Determine validity of IMU	Moderately high correlations between criterion and IMU (r = 0.67–0.69); Underestimation of jump height (9.2–10.0 cm/19.8–21.0%)	Caution in measuring jumps due to underestimation and low sensitivity to detect changes.
Piatti et al., 2022 [49]	Describe the frequency and intensity of jumps in playing positions in a season.	Differences between playing positions (95% CI); +Frequency of jumps in training—matches; +Intensity in matches (95% CI).	Usefulness for external load control, individualization and specificity of training.
Schleitzer, 2022 [50]	Determine validity and reliability of IMU on sand surfaces	Jump detection accuracy (100/97.5%); Height validity (ICC = 0.937/0.946).	Utility for external load control of sand training.
Schmidt, 2021 [51]	Determine validity and reliability of IMU on sand surfaces	Excellent accuracy (0.975) for counting jumps and good to excellent correlations for blocking (r = 0.81) and spiked jumps (r = 0.90).	Usefulness for external load control and jump evaluation in beach volleyball.
Setuain, 2021 [52]	To evaluate vertical jump mechanics before and after a controlled load (volume and intensity) of a training session	A 10% decrease in post-training vertical ground reaction force was observed (*p* = 0.02).	Useful for controlling fatigue through jumping ability.
Skazalski, 2018 [24]	Determine validity and reliability of IMU	Counting accuracy (99.3%); Overestimation of jump (5.5 cm, 12% of average height).	Utility for external load control; caution in assessing jump heights.
Skazalski, 2018b [53]	Compare jumps and playing positions	Setters performed more jumps; Opposites more high intensity jumps.	Usefulness for control and individualization of external training load.
Vlantes, 2017 [54]	Describe internal and external loads and relate them to each other	Differences between playing positions in internal and external load (*p* < 0.01); Difference between sets of matches (*p* < 0.05).	Usefulness for individualization of training load.

**Table 5:** Validity characteristics of the found studies.

Study	Environment	Instrument 1 (Criterion)	Variables Measured	Instrument 2 (Validated)	Type of Analysis	Value
Jarning, 2015 [41]	Structured practice (n = 1)	Observers (n = 1)	4 specific jumps and 3 movements without jumps	ActiGraph GT3X+	Anova	>0.05
Gageler, 2015 [39]	Training (n = 1)	Observers (n = 1)	Movements with jumps * and without jumps	GPSports Systems	Instrument 1 (n=)	1201
					Instrument 2 (n=)	1198
					True+	114 (95%)
					False+	54 (4%)
					False−	57 (5%)
Charlton et al., 2017 [10]	Structured practice; (n = 1) Training (n = 1); Competition (n = 1)	Observers (n = 2); Intra-obs (k = 0.953)	Jumps *	Vert Classic	Instrument 1 (n=)	1487
					Instrument 2 (n=)	1307
					False+	2
					False−	180
					Precision	0.998
					Recall	0.879
McDonald, 2017 [47]	Structured practice (n = 1)	Observers (n = 1) Blinded	6 specific jumps and 6 non-jumping movements	Vert Classic	Instrument 1 (n=)	728
					Instrument 2 (n=)	705
					Sensitivity	96.80%
					Specificity	100%
					Positive predictive value	100%
					Negative predictive value	94%
					Mean difference	−2 (−4.3 a 0.2)
					LOA	−9.0 a 5.0
					ME	0.70%
					% ME	0.1%
	Competition (n = 1)	Observers (n = 1) Blinded	Jumps > 15cm	Vert Classic	Instrument 1 (n=)	977
					Instrument 2 (n=)	1032
					Difference of means	5 (0.7 a 8.5)
					LOA	−8 a 17
Skazalski, 2018 [24]	Trainings (n = 3); Matches (n = 2)	Observers (n = 2) Blinded	Jumps *	Vert Classic	Instrument 1 (n=)	3637
					Instrument 2 (n=)	3612
					False+	12
					False−	25
Schleitzer, 2022 [50]	Structured and unstructured practice (n = 1)	Observers (n = 1)	block, attack, serve	Suunto movesense original	Instrument 1 (n=)	319
					Instrument 2 (n=)	306
					True+	306 (95.9%)
					False−	13 (4.1%)
					False +	14 (4.4%)
	Laboratory (n = 3)	Observers (n = 1)	CMJ	Suunto movesense original	Instrument 1 (n=)	200
					Instrument 2 (n=)	200
					True+ (%)	100
					False− (%)	0
					Instrument 1 (n=)	
Schmidt, 2021 [51]	Competition (n = 2–4/1 set)	Observers (n = 2)	Spike, block, serve, set, other	Vert TM Classic	Instrument 1	439
					Instrument 2	392
					False+	10
					False−	47
					Precision	0.975
					Recall	0.893

* Any occasion when both feet of an athlete cease to have contact with the ground.

**Table 6:** Reliability characteristics of the studies found.

Study	Instrument 1 (Criterion)	Instrument 2 (Validated)	Logarithm	Variables Measured	Type of Analysis	Value
Gageler, 2015 [39]	Force platform (Kistler 9287BA) 1000 Hz	GPSports Systems	JH = gravity × ToF^2^/8	Blocks, spikes	Mean error (s).	−0.015 ± 0.058
Borges, 2017 [36]	VERTEC (Sports Imports, USA)	Vert Classic	Not specified	Jumping, blocking, attacking	Instrument 1 (cm);	70.9 ±8.2
					Instrument 2 (cm);	76.3 ±7.5
					Pearson’s r;	0.75
					Standard error (cm);	5.3 (4.8 a 6.0)
					Coefficient of variation.	7.80%
Charlton et al., 2017 [10]	3D analysis (Vicon, Oxford, UK) 250 Hz;	Vert Classic 1	Not specified	set, spike, block and serve	Pearson’s r;	0.83
					Mean bias (cm).	3.57
	3D analysis (Vicon, Oxford, UK) 250 Hz	Vert Classic 2	Not specified	set, spike, block and serve	Pearson’s r;	0.97
					Mean bias (cm).	4.28
	Vert Classic 1	Vert Classic 2	Not specified	set, spike, block and serve	Pearson’s r;	0.96–0.99
					Mean bias (cm);	−0.83
					LoA (cm).	−4.55–2.89
McDonald, 2017 [47]	3D analysis (Motion Analysis, Rohnert Park, CA, USA) 240 Hz	Vert Classic	Not specified	Maximum and sybmaximum jumps with 1 and 2 hands	Difference in means (cm);	2.5 (−4.7 a 9.7)
					ME (cm);	2.6
					% ME.	4.40%
Skazalski, 2018 [24]	Vertec (Sports Imports, USA)	Vert Classic 1	Not specified	Jumps with 1 and 2 hands and with 2–3 steps	CCI;	0.85 (0.80 to 0.89)
					MDC (cm);	9.7
					Error (cm).	5.5 (4.5 to 6.5)
	Force platform (ForceDecks, NMP)	Vert Classic1	Not specified	CMJ	CCI;	0.93 (0.89 to 0.96)
					MDC (cm);	5.5
					Error (cm).	9.1 (8.1 to 10)
	Vert Classic1	Vert Classic2	Not specified	Jumps with 1 and 2 hands and with 2–3 steps	CCI;	0.99 (0.98 to 0.99)
					MDC (cm);	2.3
					Error (cm).	−0.3 (−0.6 to 0.0)
Montoye, 2018 [48]	Vertec (Sports Imports, USA)	Blast athletic performance	Not specified	CMJ with arms; CMJ with arms and a previous step.	Pearson’s r	r = 0.68
					Mean absolute error (cm);	9.1 (8.5 to 9.5)
					Error %.	19.9
Schleitzer, 2022 [50]	Force platform (9287C, Switzerland) 1500 Hz	Suunto movesense original	$\mathrm{Jump}\;\mathrm{height}\;h=9.81\frac{m}{s^{2}}\cdot \frac{t^{2}}{8}$	CMJ	Blas;	−1.44
					LoA-;	−7.17
					LoA+;	4.29
					ICC;	0.866 (0.817–0.902)
					Pearson’s r.	0.866 (0.807–0.908), *p* < 0.001
Markovic, 2021 [46]	Force platform (AMTI. USA) 1000 Hz	Personalizado	$h=\frac{t_{F}{}^{2}\;\cdot g_{0}}{8}$	SJ CMJ	Blas (cm);	−0.18 (−0.6; 0.24) CMJ
					LoA− (cm);	−2.26 (−2.99; −1.54) CMJ
					LoA+ (cm);	1.9 (1.17; 2.63)
					ICC;	0.975 (0.944; 0.989)
					*t*-test (t, *p*, d);	(0.897, 0.379, 0.176)
					McV (%).	1.896
Damji, 2021 [37]	Shimmer3	G-Vert	Not specified	Maximum and sub-maximum CMJ	Limit of agreement %;	−84.13 y 52.37
					Mean bias %;	−15.88
					Confidence interval;	−35.99% a 4.23%
					ICC;	0.49
					CCC.	0.37
Schmidt, 2021 [51]	Force platform (AMTI. USA) 1000 Hz	VertTM	Not specified	Spike, block	Typical error estimate (cm);	3.02–3.13
					Mean bias (cm);	2.61–7.69
					LoA (cm).	7.65–6.60

## Data Availability

The datasets generated from the study are available from the corresponding author on reasonable request.

## References

[1] Arlotti J.S., Carroll W.O., Afifi Y., Talegaonkar P., Albuquerque L., Burch V.B.F., Ball J.E., Chander H., Petway A. (2022). Benefits of IMU-based Wearables in Sports Medicine: Narrative Review. Int. J. Kinesiol. Sport. Sci..

[2] Camomilla V., Bergamini E., Fantozzi S., Vannozzi G. (2018). Trends Supporting the In-Field Use of Wearable Inertial Sensors for Sport Performance Evaluation: A Systematic Review. Sensors.

[3] Boyd L.J., Ball K., Aughey R.J. (2013). Quantifying external load in Australian football matches and training using accelerometers. Int. J. Sport. Physiol. Perform..

[4] Sato K., Smith S.L., Sands W.A. (2009). Validation of an accelerometer for measuring sport performance. J. Strength Cond. Res..

[5] Lomond K.V., Turcotte R.A., Pearsall D.J. (2007). Three-dimensional analysis of blade contact in an ice hockey slap shot, in relation to player skill. Sport. Eng..

[6] Gabbett T.J. (2015). Relationship Between Accelerometer Load, Collisions, and Repeated High-Intensity Effort Activity in Rugby League Players. J. Strength Cond. Res..

[7] Wellman A.D., Coad S.C., Goulet G.C., McLellan C.P. (2016). Quantification of Competitive Game Demands of NCAA Division I College Football Players Using Global Positioning Systems. J. Strength Cond. Res..

[8] Giandolini M., Poupard T., Gimenez P., Horvais N., Millet G.Y., Morin J.B., Samozino P. (2014). A simple field method to identify foot strike pattern during running. J. Biomech..

[9] Polglaze T., Dawson B., Hiscock D.J., Peeling P. (2015). A comparative analysis of accelerometer and time-motion data in elite men’s hockey training and competition. Int. J. Sport. Physiol. Perform..

[10] Charlton P.C., Kenneally-Dabrowski C., Sheppard J., Spratford W. (2017). A simple method for quantifying jump loads in volleyball athletes. J. Sci. Med. Sport..

[11] Malone J.J., Lovell R., Varley M.C., Coutts A.J. (2017). Unpacking the Black Box: Applications and Considerations for Using GPS Devices in Sport. Int. J. Sport. Physiol. Perform..

[12] Bonaiuto V., Boatto P., Lanotte N., Romagnoli C., Annino G. (2018). A Multiprotocol Wireless Sensor Network for High Performance Sport Applications. Appl. Syst. Innov..

[13] Van der Kruk E., Reijne M.M. (2018). Accuracy of human motion capture systems for sport applications; state-of-the-art review. Eur. J. Sport. Sci..

[14] Connor S.O., McCaffrey N., Whyte E., Moran K. (2016). The novel use of a SenseCam and accelerometer to validate training load and training information in a self-recall training diary. J. Sport. Sci..

[15] Gomes B.B., Ramos N.V., Conceição F.A.V., Sanders R.H., Vaz M.A., Vilas-Boas J.P. (2015). Paddling Force Profiles at Different Stroke Rates in Elite Sprint Kayaking. J. Appl. Biomech..

[16] Lee J.B., Mellifont R.B., Burkett B.J. (2010). The use of a single inertial sensor to identify stride, step, and stance durations of running gait. J. Sci. Med. Sport..

[17] Garrett J., Graham S.R., Eston R.G., Burgess D.J., Garrett L.J., Jakeman J., Norton K. (2019). A Novel Method of Assessment for Monitoring Neuromuscular Fatigue in Australian Rules Football Players. Int. J. Sport. Physiol. Perform..

[18] Budig M., Höltke V., Keiner M. (2019). Accuracy of optical heart rate measurement and distance measurement of a fitness tracker and their consequential use in sports. Ger. J. Exerc. Sport. Res..

[19] Rago V., Brito J., Figueiredo P., Costa J., Barreira D., Krustrup P., Rebelo A. (2020). Methods to collect and interpret external training load using microtechnology incorporating GPS in professional football: A systematic review. Res. Sport. Med..

[20] Praz C., Fasel B., Vuistiner P., Aminian K., Kayser B. (2016). Optimal slopes and speeds in uphill ski mountaineering: A laboratory study. Eur. J. Appl. Physiol..

[21] Kyprianou E., Di Salvo V., Lolli L., Al Haddad H., Villanueva A.M., Gregson W., Matthew W. (2022). To Measure Peak Velocity in Soccer, Let the Players Sprint. J. Strength Cond. Res..

[22] Roell M., Roecker K., Gehring D., Mahler H., Gollhofer A. (2018). Player Monitoring in Indoor Team Sports: Concurrent Validity of Inertial Measurement Units to Quantify Average and Peak Acceleration Values. Front. Physiol..

[23] García-de-Alcaraz A., Ramírez-Campillo R., Rivera-Rodríguez M., Romero-Moraleda B. (2020). Analysis of jump load during a volleyball season in terms of player role. J. Sci. Med. Sport..

[24] Skazalski C., Whiteley R., Bahr R. (2018). High jump demands in professional volleyball-large variability exists between players and player positions. Scand. J. Med. Sci. Sport..

[25] Lima R.F., Palao J.M., Clemente F.M. (2019). Jump Performance During Official Matches in Elite Volleyball Players: A Pilot Study. J. Hum. Kinet..

[26] Rantalainen T., Finni T., Walker S. (2020). Jump height from inertial recordings: A tutorial for a sports scientist. Scand. J. Med. Sci. Sport..

[27] Brooks E.R., Benson A.C., Bruce L.M. (2018). Novel technologies found to be valid and reliable for the measurement of vertical jump height with jump-and-reach testing. J. Strength Cond. Res..

[28] Oliveira L.D.S., Moura T.B.M.A., Rodacki A.L.F., Tilp M., Okazaki V.H.A. (2020). A systematic review of volleyball spike kinematics: Implications for practice and research. Int. J. Sport. Sci. Coach..

[29] Fuchs P.X., Menzel H.J.K., Guidotti F., Bell J., von Duvillard S.P., Wagner H. (2019). Spike jump biomechanics in male versus female elite volleyball players. J. Sport. Sci..

[30] Clemente F., Badicu G., Hasan U.C., Akyildiz Z., Pino-Ortega J., Silva R., Rico-González M. (2022). Validity and reliability of inertial measurement units for jump height estimations: A systematic review. Hum. Mov..

[31] Ato M., López J.J., Benavente A. (2013). Un sistema de clasificación de los diseños de investigación en psicología. Anales de Psicología.

[32] Page M.J., McKenzie J.E., Bossuyt P.M., Boutron I., Hoffmann T.C., Mulrow C.D., Shamseer L., Tetzlaff J.M., Akl E.A., Brennan S.E. (2021). The PRISMA 2020 statement: An updated guideline for reporting systematic reviews. BMJ.

[33] Rico-González M., Pino-Ortega J., Clemente F., Arcos A.L. (2021). Guidelines for performing systematic reviews in sports science. Biol. Sport..

[34] Moher D., Shamseer L., Clarke M., Ghersi D., Liberati A., Petticrew M., Shekelle P., Stewart L.A., PRISMA-P Group (2015). Preferred reporting items for systematic review and meta-analysis protocols (PRISMA-P) 2015 statement. Syst. Rev..

[35] O’Reilly M., Caulfield B., Ward T., Johnston W., Doherty C. (2018). Wearable Inertial Sensor Systems for Lower Limb Exercise Detection and Evaluation: A Systematic Review. Sport. Med..

[36] Borges T.O., Moreira A., Bacchi R., Finotti R.L., Ramos M., Lopes C.R., Aoki M.S. (2017). Validation of the VERT wearable jump monitor device in elite youth volleyball players. Biol. Sport.

[37] Damji F., MacDonald K., Hunt M.A., Taunton J., Scott A. (2021). Using the VERT wearable device to monitor jumping loads in elite volleyball athletes. PLoS ONE.

[38] De Leeuw A.W., van der Zwaard S., van Baar R., Knobbe A. (2022). Personalized machine learning approach to injury monitoring in elite volleyball players. Eur. J. Sport. Sci..

[39] Gageler H.W., Wearing S., James A.D. (2015). Automatic jump detection method for athlete monitoring and performance in volleyball. Int. J. Perform. Anal. Sport.

[40] Gielen J., Mehuys E., Berckmans D., Meeusen R., Aerts J.M. (2022). Monitoring Internal and External Load During Volleyball Competition. Int. J. Sport. Physiol. Perform..

[41] Jarning J.M., Mok K.M., Hansen B.H., Bahr R. (2015). Application of a tri-axial accelerometer to estimate jump frequency in volleyball. Sport. Biomech..

[42] João P.V., Medeiros A., Ortigão H., Lee M., Mota M.P. (2021). Global position analysis during official elite female beach volleyball competition: A pilot study. Appl. Sci..

[43] Kupperman N., Curtis M.A., Saliba S.A., Hertel J. (2021). Quantification of Workload and Wellness Measures in a Women’s Collegiate Volleyball Season. Front. Sport. Act. Living.

[44] Lima R.F., Andrés J.M.P., Castro H., Clemente F. (2019). Measuring the training external jump load of elite male volleyball players: An exploratory study in Portuguese League. Retos Nuevas Tend. En Educ. Física Deporte Y Recreación.

[45] Lima R.F., Silva A., Afonso J., Castro H., Clemente F.M. (2020). External and internal Load and their Effects on Professional Volleyball Training. Int. J. Sport. Med..

[46] Marković S., Dopsaj M., Tomažič S., Kos A., Nedeljković A., Umek A. (2021). Can IMU Provide an Accurate Vertical Jump Height Estimate?. Appl. Sci..

[47] MacDonald K., Bahr R., Baltich J., Whittaker J.L., Meeuwisse W.H. (2017). Validation of an inertial measurement unit for the measurement of jump count and height. Phys. Ther. Sport..

[48] Montoye A.H., Mitrzyk J. (2019). Validity of the blast athletic performance monitor for assessing vertical jump height in female volleyball players. Meas. Phys. Educ. Exerc. Sci..

[49] Piatti M., Ambrosi E., Dedda G., Omeljaniuk R.J., Turati M., Bigoni M., Gaddi D. (2022). Jump performance during a season in elite volleyball players. J. Sport. Med. Phys. Fit..

[50] Schleitzer S., Wirtz S., Julian R., Eils E. (2022). Development and evaluation of an inertial measurement unit (IMU) system for jump detection and jump height estimation in beach volleyball. Ger. J. Exerc. Sport Res..

[51] Schmidt M., Meyer E., Jaitner T. (2021). Quantifying jump-specific loads in beach volleyball by an inertial measurement device. Int. J. Sport. Sci. Coach..

[52] Setuain I., Berriel G.P., Lanz D., Schons P., Oliveira H.B., Grazioli R., Peyré-Tartaruga L.A., Garcia-Tabar I., Cadore E.L. (2022). Jump performance and mechanics after a regular training bout in elite volleyball players. J. Sport. Med. Phys. Fit..

[53] Skazalski C., Whiteley R., Hansen C., Bahr R. (2018). A valid and reliable method to measure jump-specific training and competition load in elite volleyball players. Scand. J. Med. Sci. Sport..

[54] Vlantes T.G., Readdy T. (2017). Using Microsensor Technology to Quantify Match Demands in Collegiate Women’s Volleyball. J. Strength Cond. Res..

[55] Slim K., Nini E., Forestier D., Kwiatkowski F., Panis Y., Chipponi J. (2003). Methodological index for non-randomized studies (minors): Development and validation of a new instrument. ANZ J. Surg..

[56] Kim S.Y., Park J.E., Lee Y.J., Seo H.J., Sheen S.S., Hahn S., Jang B.H., Son H.J. (2013). Testing a tool for assessing the risk of bias for nonrandomized studies showed moderate reliability and promising validity. J. Clin. Epidemiol..

[57] Jiménez-Olmedo J.M., Penichet-Tomás A. (2015). Injuries and pathologies in beach volleyball players: A systematic review. J. Hum. Sport Exerc..

[58] Pereira L.A., Freitas T.T., Marín-Cascales E., Bishop C., McGuigan M.R., Loturco I. (2021). Effects of training on sand or hard surfaces on sprint and jump performance of team-sport players: A systematic review with meta-analysis. Strength Cond. J..

[59] Huang J., Gates A.J., Sinatra R., Barabási A.L. (2020). Historical comparison of gender inequality in scientific careers across countries and disciplines. Proc. Natl. Acad. Sci. USA.

[60] Pawlik D., Mroczek D. (2022). Fatigue and Training Load Factors in Volleyball. Int. J. Environ. Res. Public Health.

[61] De Leeuw A.W., van Baar R., Knobbe A., van der Zwaard S. (2022). Modeling Match Performance in Elite Volleyball Players: Importance of Jump Load and Strength Training Characteristics. Sensors.

[62] Heishman A.D., Curtis M.A., Saliba E., Hornett R.J., Malin S.K., Weltman A.L. (2018). Noninvasive Assessment of Internal and External Player Load: Implications for Optimizing Athletic Performance. J. Strength Cond. Res..

[63] Hughes T., Jones R.K., Starbuck C., Sergeant J.C., Callaghan M.J. (2019). The value of tibial mounted inertial measurement units to quantify running kinetics in elite football (soccer) players. A reliability and agreement study using a research orientated and a clinically orientated system. J. Electromyogr. Kinesiol..

[64] Daly E., Esser P., Griffin A., Costello D., Servis J., Gallagher D., Ryan L. (2022). Development of a Novel Coaching Platform to Improve Tackle Technique in Youth Rugby Players: A Proof of Concept. Sensors.

[65] Bahr M.A., Bahr R. (2014). Jump frequency may contribute to risk of jumper’s knee: A study of interindividual and sex differences in a total of 11,943 jumps video recorded during training and matches in young elite volleyball players. Br. J. Sport. Med..

[66] Thomas J.R., Martin P., Etnier J., Silverman S.J. (2022). Research Methods in Physical Activity.

[67] Anguera M.T., Hernández-Mendo A. (2016). Advances in mixed methods observational studies in sports sciences. Cuadernos de Psicología del Deporte.

[68] Buckthorpe M., Morris J., Folland J.P. (2012). Validity of vertical jump measurement devices. J. Sport. Sci..

[69] Petrigna L., Karsten B., Marcolin G., Paoli A., D’Antona G., Palma A., Bianco A. (2019). A Review of Countermovement and Squat Jump Testing Methods in the Context of Public Health Examination in Adolescence: Reliability and Feasibility of Current Testing Procedures. Front. Physiol..

[70] Hopkins W.G. (2000). Measures of reliability in sports medicine and science. Sport. Med..

[71] Soler-López A., García-de-Alcaraz A., Moreno-Villanueva A., Pino-Ortega J. (2022). Concurrent Validity and Reliability of Devices to Measure Jump Height in Men’s Handball Players. Sensors.

[72] Özdemir A.T., Barshan B. (2014). Detecting falls with wearable sensors using machine learning techniques. Sensors.

[73] Xu Q., Wang L., Younis M.I. (2022). Multi-Threshold Inertial Switch with Acceleration Direction Detection Capability. IEEE Trans. Ind. Electron..

